# Quadratic transformation inequalities for Gaussian hypergeometric function

**DOI:** 10.1186/s13660-018-1848-y

**Published:** 2018-09-21

**Authors:** Tie-Hong Zhao, Miao-Kun Wang, Wen Zhang, Yu-Ming Chu

**Affiliations:** 1 Department of Mathematics, Hangzhou Normal University, Hangzhou, China; 2 Department of Mathematics, Huzhou University, Huzhou, China; 3 Friedman Brain Institute, Icahn School of Medicine at Mount Sinai, New York, New York, USA

**Keywords:** 33C05, 26D20, Hypergeometric function, Quadratic transformation, Generalized Grötzsch ring function, Duplication inequality

## Abstract

In the article, we present several quadratic transformation inequalities for Gaussian hypergeometric function and find the analogs of duplication inequalities for the generalized Grötzsch ring function.

## Introduction

The Gaussian hypergeometric function $_{2}F_{1}(a,b;c;x)$ with real parameters $a,b$, and *c* $(c\neq0,-1,-2,\dots)$ is defined by [1, 4, 24, 41]

$$ F(a,b;c;x)=_{2}F_{1}(a,b;c;x)=\sum _{n=0}^{\infty}\frac {(a,n)(b,n)}{(c,n)}\frac{x^{n}}{n!} $$

for $x\in(-1,1)$, where $(a,n)=a(a+1)(a+2)\cdots(a+n-1)$ for $n=1,2,\dots$, and $(a,0)=1$ for $a\neq0$. The function $F(a,b;c;x)$ is called zero-balanced if $c=a+b$. The asymptotical behavior for $F(a,b;c;x)$ as $x\rightarrow1$ is as follows (see [4, Theorems 1.19 and 1.48])

1.1 $$ \textstyle\begin{cases} F(a,b;c;1)=\Gamma(c)\Gamma(c-a-b)/[\Gamma(c-a)\Gamma(c-b)],&a+b< c,\\ B(a,b)F(a,b;c;z)+\log(1-z)=R(a,b)+O((1-z)\log(1-z)),&a+b=c,\\ F(a,b;c;z)=(1-z)^{c-a-b}F(c-a,c-b;c;z),&a+b>c, \end{cases} $$

where $\Gamma(x)=\int_{0}^{\infty}t^{x-1}e^{-t}\,dt$ [10, 25, 43, 44, 47] and $B(p,q)=[{\Gamma(p)\Gamma(q)}]/[{\Gamma(p+q)}]$ are the classical gamma and beta functions, respectively, and

1.2 $$ R(a,b)=-\psi(a)-\psi(b)-2\gamma,\qquad R \biggl(\frac{1}{4}, \frac{3}{4} \biggr)=\log64, $$

$\psi(z) =\Gamma'(z)/\Gamma(z)$), and $\gamma=\lim_{n\rightarrow \infty} (\sum_{k=1}^{n}{1}/{k}-\log n )=0.577\dots$ is the Euler–Mascheroni constant [21, 50].

As is well known, making use of the hypergeometric function, Branges proved the famous Bieberbach conjecture in 1984. Since then, $F(a,b;c;x)$ and its special cases and generalizations have attracted attention of many researchers, and was studied deeply in various fields [2, 5, 9, 11–18, 20, 22, 23, 26, 30, 31, 35–37, 40, 45, 46, 48]. A lot of geometrical and analytic properties, and inequalities of the Gaussian hypergeometric function have been obtained [3, 6–8, 19, 29, 32, 34, 38, 49].

Recently, in order to investigate the Ramanujan’s generalized modular equation in number theory, Landen inequalities, Ramanujan cubic transformation inequalities, and several other quadratic transformation inequalities for zero-balanced hypergeometric function have been proved in [27, 28, 32, 39, 42]. For instance, using the quadratic transformation formula [24, (15.8.15), (15.8.21)]

1.3 $$ F \biggl(\frac{1}{4},\frac{3}{4};1;\frac{8r(1+r)}{(1+3r)^{2}} \biggr)=\sqrt {1+3r}F \biggl(\frac{1}{4},\frac{3}{4};1;r^{2} \biggr), $$

Wang and Chu [32] found the maximal regions of the $(a,b)$-plane in the first quadrant such that inequality

1.4 $$ F \biggl(a,b;a+b;\frac{8r(1+r)}{(1+3r)^{2}} \biggr)\leq \sqrt{1+3r}F \bigl(a,b;a+b;r^{2}\bigr) $$

or its reversed inequality

1.5 $$ F \biggl(a,b;a+b;\frac{8r(1+r)}{(1+3r)^{2}} \biggr)\geq \sqrt{1+3r}F \bigl(a,b;a+b;r^{2}\bigr) $$

holds for each $r\in(0,1)$. Moreover, very recently in [33], some Landen-type inequalities for a class of Gaussian hypergeometric function $_{2}F_{1} (a,b;(a+b+1)/2;x )\ (a,b>0)$, which can be viewed as a generalization of Landen identities of the complete elliptic integrals of the first kind

$$ F \biggl(\frac{1}{2},\frac{1}{2};1;\frac{4r}{(1+r)^{2}} \biggr)=(1+r)F \biggl(\frac{1}{2},\frac{1}{2};1;r^{2} \biggr), $$

have also been proved. As an application, the analogs of duplication inequalities for the generalized Grötzsch ring function with two parameters [33]

1.6 $$ \mu_{a,b}(r)=\frac{B(a,b)}{2}\frac{ F (a,b;(a+b+1)/2;1-r^{2} )}{F (a,b;(a+b+1)/2;r^{2} )},\quad r \in(0,1), $$

have been derived. In fact, the authors have proved

### Theorem 1.1

*For* $(a,b)\in\{(a,b)|a,b>0,ab\geq a+b-10/9, a+b\geq2\}$, *let* $x=x(r)=2\sqrt{r}/(1+r)$, *then the Landen*-*type inequality*

1.7 $$ \bigl(xx'\bigr)^{(a+b-1)/2} F \biggl(a,b; \frac{a+b+1}{2};x^{2} \biggr)>(1+r) \bigl(rr' \bigr)^{(a+b-1)/2} F \biggl(a,b;\frac{a+b+1}{2};r^{2} \biggr) $$

*holds for all* $r\in(0,1)$.

### Theorem 1.2

*For* $(a,b)\in\{(a,b)|a,b>0,ab\geq a+b-10/9, a+b\geq2\}$, *define the function* *g* *on* $(0,1)$ *by*

$$ g(r)=2\mu_{a,b} \biggl(\frac{2\sqrt{r}}{1+r} \biggr)-\mu_{a,b}(r). $$

*Then* *g* *is strictly increasing from* $(0,1)$ *onto* $(-\infty,0)$. *In particular*, *the inequality*

$$ 2\mu_{a,b} \biggl(\frac{2\sqrt{r}}{1+r} \biggr)< \mu_{a,b}(r) $$

*holds for each* $r\in(0,1)$ *with* $(a,b)\in\{(a,b)|a,b>0,ab\geq a+b-10/9, a+b\geq2\}$.

The purpose of this paper is to establish several quadratic transformation inequalities for Gaussian hypergeometric function $_{2}F_{1}(a,b;(a+b+1)/2;x)$ $(a,b>0)$, such as inequalities (1.4), (1.5) and (1.7), and thereby prove the analogs of Theorem 1.2.

We recall some basic facts about $\mu_{a,b}(r)$ (see [33]). The limiting values of $\mu_{a,b}(r)$ at 0 and 1 are

1.8 $$\begin{aligned} \mu_{a,b}\bigl(0^{+}\bigr)={}&\lim_{r\rightarrow0^{+}} \frac{B(a,b)}{2}F \biggl(a,b;\frac {a+b+1}{2};1-r^{2} \biggr) \\ ={}&\textstyle\begin{cases}\frac{B(a,b)}{2}H(a,b), &a+b< 1,\\ +\infty,&a+b\geq1, \end{cases}\displaystyle \end{aligned}$$

1.9 $$\begin{aligned} \mu_{a,b}\bigl(1^{-}\bigr)={}&\lim_{r\rightarrow1^{-}} \frac{B(a,b)}{2 F (a,b;\frac {a+b+1}{2};r^{2} )} =\textstyle\begin{cases}\frac{B(a,b)}{2H(a,b)}, &a+b< 1,\\ 0,&a+b\geq1, \end{cases}\displaystyle \end{aligned}$$

and the derivative formula of $\mu_{a,b}(r)$ is

1.10 $$ \frac{d\mu_{a,b}(r)}{dr}=-\frac{{\Gamma(\frac{a+b+1}{2})}^{2}}{\Gamma (a+b)}\frac{1}{r^{a+b}{r'}^{a+b+1}{F (a,b;(a+b+1)/2;r^{2} )}^{2}}. $$

Here and in what follows,

$$H(a,b)=\frac{B(\frac{a+b+1}{2},\frac{1-a-b}{2})}{B(\frac{1+b-a}{2},\frac {1+a-b}{2})}. $$

## Lemmas

In order to prove our main results, we need several lemmas, which we present in this section. Throughout this section, we denote

2.1 $$ F(x)=F \biggl(a,b,\frac{a+b+1}{2};x \biggr),\qquad G(x)=F \biggl(a+1,b+1;\frac {a+b+3}{2};x \biggr) $$

for $(a,b)\in(0,+\infty)\times(0,+\infty)\setminus\{p,q\}$ with $p=(1/4, 3/4)$ and $q=(3/4, 1/4)$, and

2.2 $$ \widehat{F}(x)= \biggl(\frac{1}{4},\frac{3}{4};1;x \biggr),\qquad \widehat {G}(x)=F \biggl(\frac{5}{4},\frac{7}{4};2;x \biggr). $$

**Figure 1:**
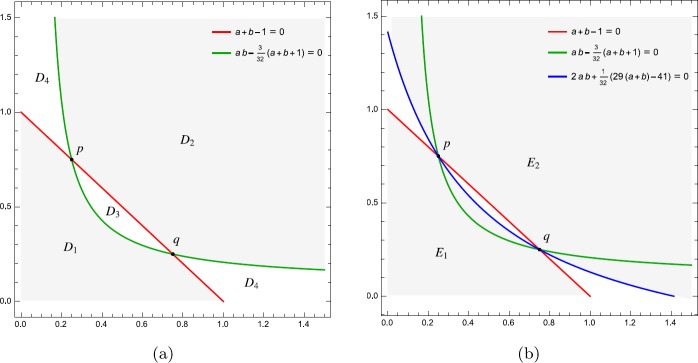
The regions $D_{i}$ for $i=1,2,3,4$, $E_{1},E_{2}$ and their boundary curves

For the convenience of readers, we introduce some regions in $\{(a,b)\in \mathbb{R}^{2}| a>0,b>0\}$ and refer to Fig. 1 for illustration:

$$\begin{aligned} &D_{1}= \biggl\{ (a,b)\Big| a,b>0, a+b\leq1,ab-\frac{3(a+b+1)}{32}\leq0 \biggr\} , \\ &D_{2}= \biggl\{ (a,b)\Big| a,b>0, a+b\geq1,ab-\frac{3(a+b+1)}{32}\geq0 \biggr\} , \\ &D_{3}= \biggl\{ (a,b)\Big| a,b>0, a+b< 1,ab-\frac{3(a+b+1)}{32}>0 \biggr\} , \\ &D_{4}= \biggl\{ (a,b)\Big| a,b>0, a+b>1,ab-\frac{3(a+b+1)}{32}< 0 \biggr\} , \\ &E_{1}= \biggl\{ (a,b)\Big| a,b>0, a+b\leq1,2ab+\frac{29(a+b)-41}{32}\leq0 \biggr\} , \\ &E_{2}= \biggl\{ (a,b)\Big| a,b>0, a+b\geq1,2ab+\frac{29(a+b)-41}{32}\geq0 \biggr\} . \end{aligned}$$

Obviously, $\bigcup_{i=1}^{4}D_{i}=(0,+\infty)\times(0,+\infty)$ and $D_{i}\cap D_{j}=\emptyset$ for $i\neq j\in\{1,2,3,4\}$ except that $D_{1}\cap D_{2}=\{p,q\}$. Moreover, $D_{1}\subset E_{1}$ and $D_{2}\subset E_{2}$.

### Lemma 2.1

([42, Theorem 2.1])

*Suppose that the power series* $f(x)=\sum_{n=0}^{\infty}a_{n}x^{n}$ *and* $g(x)=\sum_{n=0}^{\infty}b_{n}x^{n}$ *have the radius of convergence* $r>0$ *with* $b_{n}>0$ *for all* $n\in\{0,1,2,\dots\}$. *Let* $h(x)=f(x)/g(x)$ *and* $H_{f,g}=(f'/g')g-f$, *then the following statements hold true*:

1. *If the non*-*constant sequence* $\{a_{n}/b_{n}\}_{n=0}^{\infty}$ *is increasing* (*decreasing*) *for all* $n>0$, *then* $h(x)$ *is strictly increasing* (*decreasing*) *on* $(0,r)$;
2. *If the non*-*constant sequence* $\{a_{n}/b_{n}\}_{n=0}^{\infty}$ *is increasing* (*decreasing*) *for* $0< n\leq n_{0}$ *and decreasing* (*increasing*) *for* $n>n_{0}$, *then* $h(x)$ *is strictly increasing* (*decreasing*) *on* $(0,r)$ *if and only if* $H_{f,g}(r^{-})\geq(\leq) 0$. *Moreover*, *if* $H_{f,g}(r^{-})<(>) 0$, *then there exists an* $x_{0}\in(0,r)$ *such that* $h(x)$ *is strictly increasing* (*decreasing*) *on* $(0,x_{0})$ *and strictly decreasing* (*increasing*) *on* $(x_{0},r)$.

### Lemma 2.2

1. *The function* $\eta(x)=F(x)/\widehat{F}(x)$ *is strictly decreasing on* $(0,1)$ *if* $(a,b)\in D_{1}\setminus\{p,q\}$ *and strictly increasing on* $(0,1)$ *if* $(a,b)\in D_{2}\setminus\{p,q\}$. *Moreover*, *if* $(a,b)\in D_{3} (\textit{or }D_{4})$, *then there exists* $\delta_{0}\in(0,1)$ *such that* $\eta(x)$ *is strictly increasing* (*decreasing*) *on* $(0,\delta _{0})$ *and strictly decreasing* (*increasing*) *on* $(\delta_{0},1)$.
2. *The function* $\widetilde{\eta}(x)=G(x)/\widehat{G}(x)$ *is strictly decreasing on* $(0,1)$ *if* $(a,b)\in E_{1}\setminus\{p,q\}$ *and strictly increasing on* $(0,1)$ *if* $(a,b)\in E_{2}\setminus\{p,q\}$. *In the remaining case*, *namely for* $x\in(0,+\infty)\times(0,+\infty )\setminus(E_{1}\cup E_{2})$, $\widetilde{\eta}(x)$ *is piecewise monotone on* $(0,1)$.

### Proof

Suppose that

$$A_{n}=\frac{(a,n)(b,n)}{ (\frac{a+b+1}{2},n )n!},\qquad A^{*}_{n}=\frac {(\frac{1}{4},n)(\frac{3}{4},n)}{(1,n)n!}, $$

then we have

2.3 $$ \eta(x)=\frac{F(x)}{\widehat{F}(x)}=\frac{\sum_{n=0}^{\infty}A_{n}x^{n}}{\sum_{n=0}^{\infty}A^{*}_{n}x^{n}}. $$

It suffices to take into account the monotonicity of $\{A_{n}/A^{*}_{n}\} _{n=0}^{\infty}$. By simple calculations, one has

2.4 $$ \frac{A_{n+1}}{A^{*}_{n+1}}-\frac{A_{n}}{A^{*}_{n}}=\frac{A_{n}\cdot\Delta _{n}}{A^{*}_{n} (\frac{a+b+1}{2} ) (\frac{1}{4}+n ) (\frac{3}{4}+n )}, $$

where

2.5 $$ \Delta_{n}= \biggl(\frac{a+b-1}{2} \biggr)n^{2}+ \biggl(ab+\frac{a+b}{2}-\frac {11}{16} \biggr)n+ab-\frac{3(a+b+1)}{32}. $$

We divide the proof into four cases.

Case 1 $(a,b)\in D_{1}\setminus\{p,q\}$. Then it follows easily that $a+b\leq1$, $ab-\frac{3(a+b+1)}{32}\leq0$ and $ab+\frac {a+b}{2}-\frac{11}{16}<0$. This, in conjunction with (2.4) and (2.5), implies that $\{A_{n}/A^{*}_{n}\}_{n=0}^{\infty}$ is strictly decreasing for all $n>0$. Therefore, (2.3) and Lemma 2.1(1) lead to the conclusion that $\eta(x)$ is strictly decreasing on $(0,1)$.

Case 2 $(a,b)\in D_{2}\setminus\{p,q\}$. Then a similar argument as in Case 1 yields $\Delta_{n}>0$ and this implies that $\eta(x)$ is strictly increasing on $(0,1)$ from (2.3), (2.4) and Lemma 2.1(1).

Case 3 $(a,b)\in D_{3}$. It follows from (2.4) and (2.5) that the sequence $\{A_{n}/A^{*}_{n}\}$ is increasing for $0\leq n\leq n_{0}$ and decreasing for $n\geq n_{0}$ for some integer $n_{0}$. Furthermore, making use of the derivative formula for Gaussian hypergeometric function

$$\frac{d F(a,b;c;x)}{dx}=\frac{ab}{c}F(a+1,b+1;c+1;x), $$

and in conjunction with (1.1) and $a+b<1$, we obtain

2.6 $$\begin{aligned} H_{F,\widehat{F}}(x)&=\frac{32ab}{3(a+b+1)} \frac{F (a+1,b+1;\frac {a+b+3}{2};x )}{F (\frac{3}{4},\frac{1}{4};2;x )}(1-x)\widehat{F}(x)-F(x) \\ &\rightarrow-H(a,b)< 0 \end{aligned}$$

as $x\rightarrow1^{-}$. Combing with (2.3), (2.6) and Lemma 2.1(2), we conclude that there exists an $x_{1}\in(0,1)$ such that $\eta(x)$ is strictly increasing on $(0,x_{1})$ and strictly decreasing on $(x_{1},1)$.

Case 4 $(a,b)\in D_{4}$. In this case, we follow a similar argument as in Case 3 and use the fact that

2.7 $$\begin{aligned} H_{F,\widehat{F}}(x)={}&\frac{32ab}{3(a+b+1)}(1-x) \frac{F (a+1,b+1;\frac{a+b+3}{2};x )}{F (\frac{3}{4},\frac {1}{4};2;x )}\widehat{F}(x)-F(x) \\ ={}&\frac{32ab}{3(a+b+1)}(1-x)^{\frac{1-a-b}{2}} \biggl[\frac{F (\frac {b-a+1}{2},\frac{a-b+1}{2};\frac{a+b+3}{2};x )}{F (\frac {3}{4},\frac{1}{4};2;x )}F \biggl(\frac{1}{4},\frac{3}{4};1;x \biggr) \\ &{}-F \biggl(\frac{b-a+1}{2},\frac{a-b+1}{2}; \frac{a+b+1}{2};x \biggr) \biggr] \\ \rightarrow{}&+\infty \end{aligned}$$

as $x\rightarrow1^{-}$ since $a+b>1$. Therefore, (2.3), (2.7) and Lemma 2.1(2) lead to the conclusion that there exists an $x_{2}\in(0,1)$ such that $\eta(x)$ is strictly decreasing on $(0,x_{2})$ and strictly increasing on $(x_{2},1)$.

Let

$$B_{n}=\frac{(a+1,n)(b+1,n)}{ (\frac{a+b+3}{2},n )n!},\qquad B^{*}_{n}=\frac {(\frac{5}{4},n)(\frac{7}{4},n)}{(2,n)n!}, $$

then we can write

2.8 $$ \widetilde{\eta}(x)=\frac{G(x)}{\widehat{G}(x)}=\frac{\sum_{n=0}^{\infty}B_{n}x^{n}}{\sum_{n=0}^{\infty}B^{*}_{n}x^{n}}. $$

Easy calculations lead to the conclusion that the monotonicity of $\{ B_{n}/B^{*}_{n}\}_{n=0}^{\infty}$ depends on the sign of

2.9 $$ \widetilde{\Delta}_{n}= \biggl(\frac{a+b-1}{2} \biggr)n^{2}+ \biggl[ab+\frac {3(a+b)}{2}-\frac{27}{16} \biggr]n+2ab+\frac{29(a+b)-41}{32}. $$

Notice that

2.10 $$\begin{aligned} H_{G,\widehat{G}}(x)&=\frac{2(a+1)(b+1)}{(a+b+3)}\cdot \frac{32F (a+2,b+2;\frac{a+b+5}{2};x )}{35F (\frac{9}{4},\frac {11}{4};3;x )}\widehat{G}(x)-G(x) \\ &=(1-x)^{-\frac{1+a+b}{2}}\omega(a,b;x), \end{aligned}$$

where

2.11 $$\begin{aligned} \omega(a,b;x)={}&\frac{64(a+1)(b+1)}{35(a+b+3)}\frac{F (\frac {b-a+1}{2},\frac{a-b+1}{2};\frac{a+b+5}{2};x )}{F (\frac {3}{4},\frac{1}{4};3;x )}F \biggl(\frac{3}{4},\frac{1}{4};2;x \biggr) \\ &{}-F \biggl(\frac{b-a+1}{2},\frac{a-b+1}{2};\frac{a+b+3}{2};x \biggr). \end{aligned}$$

It follows easily from (1.1) and (2.11) that

2.12 $$\begin{aligned} \lim_{x\rightarrow1^{-}}\omega(a,b;x)={}& \frac {64(a+1)(b+1)}{35(a+b+3)}\frac{\Gamma(\frac{a+b+5}{2})\Gamma(\frac {a+b+3}{2})}{\Gamma(a+2)\Gamma(b+2)}\frac{\Gamma(\frac{9}{4})\Gamma (\frac{11}{4})}{\Gamma(3)\Gamma(2)}\frac{\Gamma(2)\Gamma(1)}{\Gamma (\frac{5}{4})\Gamma(\frac{7}{4})} \\ &{}-\frac{\Gamma(\frac{a+b+3}{2})\Gamma(\frac{a+b+1}{2})}{\Gamma (a+1)\Gamma(b+1)} \\ ={}& \biggl(\frac{a+b-1}{2} \biggr)\frac{\Gamma(\frac{a+b+3}{2})\Gamma(\frac {a+b+1}{2})}{\Gamma(a+1)\Gamma(b+1)} \\ ={}&\textstyle\begin{cases}< 0, &a+b< 1,\\ >0, &a+b>1. \end{cases}\displaystyle \end{aligned}$$

Employing similar arguments mentioned in part (1), we obtain the desired assertions easily from (2.8)–(2.12). □

### Lemma 2.3

*Let* $D_{0}=\{(a,b)| a,b>0,a+b\geq7/4,ab\geq a+b-31/28\}$ *and* $x'=\sqrt {1-x^{2}}$ *for* $0< x<1$, *then the function*

2.13 $$ f(x)=\frac{(xx')^{\frac{a+b-1}{2}}F(a,b;\frac{a+b+1}{2};x^{2})}{F(\frac {1}{4},\frac{3}{4};1;x^{2})} $$

*is strictly increasing on* $(0,1)$ *if* $(a,b)\in D_{0}$.

### Proof

Taking the derivative of $f(x)$ yields

2.14 $$ f'(x)=\frac{(xx')^{\frac{a+b-3}{2}}}{x'F(\frac{1}{4},\frac {3}{4};1;x^{2})^{2}}f_{1}(x), $$

where

2.15 $$\begin{aligned} f_{1}(x)={}& \biggl[\frac{a+b-1}{2} \bigl(1-2x^{2}\bigr)F \biggl(a,b;\frac {a+b+1}{2};x^{2} \biggr) \\ &{}+\frac{4ab}{a+b+1}x^{2}x^{\prime 2}F \biggl(a+1,b+1;\frac{a+b+3}{2};x^{2} \biggr) \biggr]F \biggl( \frac{1}{4},\frac{3}{4};1;x^{2} \biggr) \\ &{}-\frac{3x^{2}x^{\prime 2}}{8}F \biggl(a,b;\frac{a+b+1}{2};x^{2} \biggr)F \biggl(\frac {5}{4},\frac{7}{4};2;x^{2} \biggr). \end{aligned}$$

We clearly see from (1.1) that

$$x^{\prime 2}F \biggl(\frac{5}{4},\frac{7}{4};2;x^{2} \biggr)=F \biggl(\frac{1}{4},\frac {3}{4};2;x^{2} \biggr) \leq F \biggl(\frac{1}{4},\frac{3}{4};1;x^{2} \biggr) $$

for $0< x<1$. This implies, in conjunction with (2.15), that

2.16 $$ f_{1}(x)\geq F \biggl(\frac{1}{4}, \frac{3}{4};1;x^{2} \biggr)f_{2}(x), $$

where

$$\begin{aligned} f_{2}(x)={}& \biggl[\frac{a+b-1}{2}- \biggl(a+b-\frac{5}{8} \biggr)x^{2} \biggr]F \biggl(a,b;\frac{a+b+1}{2};x^{2} \biggr) \\ &{}+\frac{4ab}{a+b+1}x^{2}\bigl(1-x^{2}\bigr)F \biggl(a+1,b+1;\frac{a+b+3}{2};x^{2} \biggr). \end{aligned}$$

It follows from the definition of hypergeometric function that

2.17 $$\begin{aligned} f_{2}(x)={}&\frac{a+b-1}{2}\sum _{n=0}^{\infty}\frac{(a,n)(b,n)}{(\frac {a+b+1}{2},n)}\frac{x^{2n}}{n!}- \biggl(a+b-\frac{5}{8} \biggr)\sum_{n=0}^{\infty}\frac{(a,n)(b,n)}{(\frac{a+b+1}{2},n)}\frac {x^{2n+2}}{n!} \\ &{}+\frac{4ab}{a+b+1} \Biggl[\sum_{n=0}^{\infty}\frac{(a+1,n)(b+1,n)}{(\frac {a+b+3}{2},n)}\frac{x^{2n+2}}{n!}-\sum_{n=0}^{\infty}\frac {(a+1,n)(b+1,n)}{(\frac{a+b+3}{2},n)}\frac{x^{2n+4}}{n!} \Biggr] \\ ={}&\frac{a+b-1}{2}+ \biggl[\frac{ab(a+b-1)}{a+b+1}- \biggl(a+b- \frac {5}{8} \biggr)+\frac{4ab}{a+b+1} \biggr]x^{2} \\ &{}+\frac{a+b-1}{2}\sum_{n=0}^{\infty}\frac {(a,n+2)(b,n+2)}{(\frac{a+b+1}{2},n+2)}\frac{x^{2n+4}}{(n+2)!} \\ &{}- \biggl(a+b-\frac{5}{8} \biggr)\sum _{n=0}^{\infty}\frac {(a,n+1)(b,n+1)}{(\frac{a+b+1}{2},n+1)}\frac{x^{2n+4}}{(n+1)!} \\ &{} +2 \Biggl[\sum_{n=0}^{\infty}\frac{(a,n+2)(b,n+2)}{(\frac {a+b+1}{2},n+2)}\frac{x^{2n+4}}{(n+1)!}-\sum_{n=0}^{\infty}\frac {(a,n+1)(b,n+1)}{(\frac{a+b+1}{2},n+1)}\frac{x^{2n+4}}{n!} \Biggr] \\ ={}&\frac{a+b-1}{2} \biggl[1-\frac{3x^{2}}{4(a+b+1)} \biggr]+ \frac {4ab(a+b-1)-4(a-b)^{2}+1}{4(a+b+1)}x^{2} \\ &{}+\sum_{n=0}^{\infty}\frac{(a,n+1)(b,n+1)}{(\frac{a+b+1}{2},n+2)} \frac {C_{n}}{(n+2)!}x^{2n+4}, \end{aligned}$$

where

2.18 $$\begin{aligned} C_{n}={}&\frac{a+b-1}{2}(a+n+1) (b+n+1)- \biggl(a+b-\frac{5}{8} \biggr) \biggl(\frac{a+b+1}{2}+n+1 \biggr) (n+2) \\ &{}+2(a+n+1) (b+n+1) (n+2)-2 \biggl(\frac{a+b+1}{2}+n+1 \biggr) (n+1) (n+2) \\ ={}& \biggl(\frac{4a+4b-7}{8} \biggr)n^{2}+ \biggl[ \frac {32ab+5(a+b)-29}{16} \biggr]n \\ &{}+\frac{4ab(a+b+3)-4(a-b)^{2}-(3a+3b+5)}{8}. \end{aligned}$$

If $(a,b)\in D_{0}$, namely, $a+b\geq7/4$ and $ab\geq a+b-31/28$, we can verify

(i) $$\begin{aligned} &4ab(a+b-1)-4(a-b)^{2}+1\\ &\quad\geq4 \biggl(a+b- \frac{31}{28} \biggr) (a+b-1)-4(a-b)^{2}+1 \\ &\quad=\frac{1}{7} \bigl[112ab-59(a+b)+38 \bigr]\geq\frac {53}{7} \biggl(a+b-\frac{86}{53} \biggr)\geq\frac{27}{28}, \end{aligned}$$
(ii) $$\begin{aligned} 32ab+5(a+b)-29&\geq32 \biggl(a+b-\frac{31}{28} \biggr)+5(a+b)-29 \\ &=\frac{37}{7} \biggl[7(a+b)-\frac{451}{259} \biggr]\geq \frac {9}{28}, \end{aligned}$$
(iii) $$\begin{aligned} &4ab(a+b+3) -4(a-b)^{2} -(3 a + 3 b + 5)\\ &\quad\geq4 \biggl(a+b-\frac{31}{28} \biggr) (a+b+3) \\ &\qquad{} -4(a-b)^{2}-(3a+3b+5)=\frac{16}{7} \bigl[7ab+2(a+b)-8 \bigr] \\ &\quad\geq \frac{16}{7} \biggl[7 \biggl(a+b-\frac{31}{28} \biggr)+2(a+b)-8 \biggr]=\frac{36}{7}\bigl[4(a+b)-7)\bigr]\geq0. \end{aligned}$$

This, in conjunction with (2.17) and (2.18), implies that $f_{2}(x)>0$ for $0< x<1$. Therefore, $f(x)$ is strictly increasing on $(0,1)$, which follows from (2.14) and (2.16) if $(a,b)\in D_{0}$. □

### Remark 2.4

The function $f(x)$ defined in Lemma 2.3 is not monotone on $(0,1)$ if two positive numbers $a,b$ satisfy $a+b<1$, since $\lim_{x\rightarrow 0^{+}}f(x)=\lim_{x\rightarrow1^{-}}f(x)=+\infty$ and Lemma 2.1(1) shows the monotonicity of $f(x)$ on $(0,1)$ if $a+b=1$. In the remaining case $a+b>1$, it follows from (2.15) that $f_{1}(0^{+})=(a+b-1)/2>0$. This, in conjunction with (2.14), implies that $f(x)$ is strictly increasing on $(0,x^{*})$ for a sufficiently small $x^{*}>0$. This enables us to find a sufficient condition for $a,b$ with $a+b>1$ such that $f(x)$ is strictly increasing on $(0,1)$ in Lemma 2.3.

The following corollary can be derived immediately from the monotonicity of $f(x)$ in Lemma 2.3 and the quadratic transformation equality (1.3).

### Corollary 2.5

*Let* $x=x(r)=\sqrt{8r(1+r)}/(1+3r)$, *if* $(a,b)\in D_{0}$, *then the inequality*

2.19 $$ \bigl(xx'\bigr)^{\frac{a+b-1}{2}}F \biggl(a,b; \frac{a+b+1}{2};x^{2} \biggr)>\sqrt {1+3r}\bigl(rr' \bigr)^{\frac{a+b-1}{2}}F \biggl(a,b;\frac{a+b+1}{2};r^{2} \biggr) $$

*holds for all* $r\in(0,1)$.

## Main results

### Theorem 3.1

*The quadratic transformation inequality*

3.1 $$ F \biggl(a,b;\frac{a+b+1}{2};\frac{8r(1+r)}{(1+3r)^{2}} \biggr)\leq \sqrt {1+3r}F \biggl(a,b;\frac{a+b+1}{2};r^{2} \biggr) $$

*holds for all* $r\in(0,1)$ *with* $a,b>0$ *if and only if* $(a,b)\in D_{1}$ *and the reversed inequality*

3.2 $$ F \biggl(a,b;\frac{a+b+1}{2};\frac{8r(1+r)}{(1+3r)^{2}} \biggr)\geq \sqrt {1+3r}F \biggl(a,b;\frac{a+b+1}{2};r^{2} \biggr) $$

*takes place for all* $r\in(0,1)$ *if and only if* $(a,b)\in D_{2}$, *with equality only for* $(a,b)=p\textit{ or }q$.

*In the remaining case* $(a,b)\in D_{3}\cup D_{4}$, *neither of the above inequalities holds for all* $r\in(0,1)$.

### Proof

Suppose that $x(r)=[8r(1+r)]/(1+3r)^{2}$, then we clearly see that $x(r)>r^{2}$ for $0< r<1$. It follows from Lemma 2.1(1) that $\eta (x(r))<\eta(r^{2})$ for $(a,b)\in D_{1}\setminus\{p,q\}$ and $\eta (x(r))>\eta(r^{2})$ for $(a,b)\in D_{2}\setminus\{p,q\}$. This, in conjunction with the quadratic transformation formula (1.3), implies

$$ F\bigl(x(r)\bigr)< \frac{\widehat{F}(x(r))}{\widehat{F}(r^{2})}F\bigl(r^{2}\bigr)=\sqrt{1+3r}F \bigl(r^{2}\bigr) $$

for $(a,b)\in D_{1}\setminus\{p,q\}$, and it degenerates to the quadratic transformation equality for $(a,b)=p (\text{or} q)$. This completes the proof of (3.1).

Inequality (3.2) can be derived analogously, and the remaining case follows easily from Lemma 2.2(1). □

### Theorem 3.2

*We define the function*

$$\varphi(r)=\sqrt{1+3\sqrt{r}}F \biggl(a,b,;\frac{a+b+1}{2};r \biggr)-F \biggl(a,b;\frac{a+b+1}{2};\frac{8\sqrt{r}(1+\sqrt{r})}{(1+3\sqrt{r})^{2}} \biggr) $$

*for* $r\in(0,1)$ *with* $a,b>0$ *and* $(a,b)\neq p,q$. *Let* $L_{1}=\{(a,b)| a+b=1, 0< a<\frac{1}{4}\textit{ or }\frac{3}{4}<a<1\}$ *and* $L_{2}=\{ (a,b)| a+b=1, \frac{1}{4}< a<\frac{3}{4}\}$. *Then the following statements hold true*:

1. *If* $(a,b)\in L_{1}(\textit{or }L_{2})$, *then* $\varphi(r)$ *is strictly increasing* (*resp*., *decreasing*) *from* $(0,1)$ *onto* $(0,[R(a,b)-\log64]/B(a,b) )$ (*resp*., $([R(a,b)-\log64]/B(a,b),0)$);
2. *If* $(a,b)\in D_{1}\setminus L_{1}$, *then* $\varphi(r)$ *is strictly increasing from* $(0,1)$ *onto* $(0,H(a,b))$;
3. *If* $(a,b)\in D_{2}\setminus L_{2}$, *then* $\varphi(r)$ *is strictly decreasing from* $(0,1)$ *onto* $(-\infty,0)$.

*As a consequence*, *the inequality*

3.3 $$\begin{aligned} F \biggl(a,b;\frac{a+b+1}{2};\frac{8r(1+r)}{(1+3r)^{2}} \biggr)&\leq \sqrt {1+3r}F \biggl(a,b,;\frac{a+b+1}{2};r^{2} \biggr) \\ & \leq F \biggl(a,b;\frac{a+b+1}{2};\frac{8r(1+r)}{(1+3r)^{2}} \biggr)+H(a,b) \end{aligned}$$

*holds for all* $r\in(0,1)$ *if* $(a,b)\in D_{1}\setminus L_{1}$, *and the following inequality is valid for all* $r\in(0,1)$:

3.4 $$\begin{aligned} & F \biggl(a,b;\frac{a+b+1}{2};\frac{8r(1+r)}{(1+3r)^{2}} \biggr) \\ &\quad \leq( \geq) \sqrt{1+3r}F \biggl(a,b,;\frac{a+b+1}{2};r^{2} \biggr) \\ &\quad\leq(\geq)F \biggl(a,b;\frac{a+b+1}{2};\frac {8r(1+r)}{(1+3r)^{2}} \biggr)+ \frac{R(a,b)-\log64}{B(a,b)} \end{aligned}$$

*if* $(a,b)\in L_{1} (\textit{resp., }L_{2})$.

### Proof

Let $z=z(r)=[8\sqrt{r}(1+\sqrt{r})]/(1+3\sqrt{r})^{2}$, then we clearly see that

3.5 $$ \frac{dz}{dr}=\frac{4(1-\sqrt{r})}{\sqrt{r}(1+3\sqrt{r})^{3}}=\frac {4(1-z)}{\sqrt{r}(1-\sqrt{r})(1+3\sqrt{r})}. $$

Taking the derivative of $\varphi(r)$ with respect to *r* and using (3.5) yields

3.6 $$\begin{aligned} \sqrt{r}(1+3\sqrt{r})\varphi'(r)={}& \frac{3\sqrt{1+3\sqrt {r}}}{4}F(r)+\sqrt{r} (\sqrt{1+3\sqrt{r}} )^{3} \frac {2ab}{a+b+1}G(r) \\ &{}-\frac{2ab}{a+b+1}\frac{4(1-z)}{1-\sqrt{r}}G(z). \end{aligned}$$

We substitute $\sqrt{r}$ for *r* in the quadratic transformation equality (1.3), then differentiate it with respect to *r* to obtain

$$ \frac{4(1-z)}{1-\sqrt{r}}\widehat{G}(z)=4\sqrt{1+3\sqrt{r}}\widehat {F}(r)+\sqrt{r} ( \sqrt{1+3\sqrt{r}} )^{3}\widehat{G}(r), $$

in other words,

3.7 $$ \frac{4(1-z)}{1-\sqrt{r}}\frac{\widehat{G}(z)}{\widehat{G}(r)}=4\sqrt {1+3\sqrt{r}} \frac{\widehat{F}(r)}{\widehat{G}(r)}+\sqrt{r} (\sqrt {1+3\sqrt{r}} )^{3}. $$

If $(a,b)\in D_{1}\setminus\{p,q\}$, then it follows from Lemma 2.2(2) that $G(x)/\widehat{G}(x)$ is strictly decreasing on $(0,1)$. This, in conjunction with $z>r$, implies that $G(z)/\widehat{G}(z)< G(r)/\widehat {G}(r)$, that is,

3.8 $$ G(z)< \frac{\widehat{G}(z)}{\widehat{G}(r)}G(r). $$

Combing (3.6), (3.7) with the inequality (3.8), we clearly see that

3.9 $$\begin{aligned} &\sqrt{r}(1+3\sqrt{r})\varphi'(r) \\ &\quad=\frac{3\sqrt{1+3\sqrt{r}}}{4}F(r)+\sqrt{r} (\sqrt{1+3\sqrt {r}} )^{3} \frac{2ab}{a+b+1}G(r)-\frac{2ab}{a+b+1}\frac {4(1-z)}{1-\sqrt{r}}G(z) \\ &\quad>\frac{3\sqrt{1+3\sqrt{r}}}{4}F(r)+\sqrt{r} (\sqrt{1+3\sqrt {r}} )^{3} \frac{2ab}{a+b+1}G(r)-\frac{2ab}{a+b+1}\frac {4(1-z)}{1-\sqrt{r}} \frac{\widehat{G}(z)}{\widehat{G}(r)}G(r) \\ &\quad=\frac{3\sqrt{1+3\sqrt{r}}}{4}F(r)+\sqrt{r} (\sqrt{1+3\sqrt {r}} )^{3} \frac{2ab}{a+b+1}G(r) \\ &\qquad{}-\frac{2ab}{a+b+1} \biggl[4\sqrt{1+3\sqrt{r}}\frac{\widehat {F}(r)}{\widehat{G}(r)}+\sqrt{r} ( \sqrt{1+3\sqrt{r}} )^{3} \biggr]G(r) \\ &\quad=4\sqrt{1+3\sqrt{r}} \biggl[\frac{3}{16}F(r)-\frac{2ab}{a+b+1} \frac {\widehat{F}(r)}{\widehat{G}(r)}G(r) \biggr] \\ &\quad =4\sqrt{1+3\sqrt{r}}\frac{F(r)^{2}}{\widehat{G}(r)} \biggl(\frac{\widehat {F}(r)}{F(r)} \biggr)'. \end{aligned}$$

It follows from Lemma 2.2(1) that $\widehat{F}(r)/F(r)$ is strictly increasing on $(0,1)$ if $(a,b)\in D_{1}\setminus\{p,q\}$. This, in conjunction with (3.9), implies that $\varphi(r)$ is strictly increasing on $(0,1)$ if $(a,b)\in D_{1}$.

Analogously, if $(a,b)\in D_{2}\setminus\{p,q\}$, then we obtain the following inequality:

$$ G(z)>\frac{\widehat{G}(z)}{\widehat{G}(r)}G(r). $$

By using a similar argument as above, we have

$$ \sqrt{r}\varphi'(r)< \frac{4F^{2}(r)}{\widehat{G}(r)} \biggl(\frac{\widehat {F}(r)}{F(r)} \biggr)'< 0, $$

since $F(r)/\widehat{F}(r)$ is strictly increasing on (0,1) if $(a,b)\in D_{2}\setminus\{p,q\}$ by Lemma 2.2(1). Hence, $\varphi(r)$ is strictly decreasing on $(0,1)$ if $(a,b)\in D_{2}$.

Notice that $\varphi(0^{+})=0$ and

3.10 $$ \lim_{r\rightarrow1^{-}}\varphi(r)=\textstyle\begin{cases} H(a,b),&a+b< 1,\\ \frac{R(a,b)-\log64}{B(a,b)},&a+b=1,\\ -\infty,&a+b>1. \end{cases} $$

Therefore, we obtain the desired assertion from (3.10). □

### Theorem 3.3

*If we define the function*

$$\phi(r)=2\mu_{a,b} \biggl(\frac{\sqrt{8r(1+r)}}{1+3r} \biggr)- \mu_{a,b}(r), $$

*for* $(a,b)\in D_{0}$, *then* $\phi(r)$ *is strictly increasing from* $(0,1)$ *onto* $(-\infty,0)$. *As a consequence*, *the inequality*

$$2\mu_{a,b} \biggl(\frac{\sqrt{8r(1+r)}}{1+3r} \biggr)< \mu_{a,b}(r) $$

*holds for all* $r\in(0,1)$ *if* $(a,b)\in D_{0}$.

### Proof

Remark 2.4 enables us to consider the case for $a+b>1$. Note that $\phi (1^{-})=0$ and

3.11 $$\begin{aligned} &\lim_{r\rightarrow0^{+}}\phi(r) \\ &\quad =\lim_{r\rightarrow0^{+}}\frac{B(a,b)}{2} \biggl[2F \biggl(a,b; \frac {a+b+1}{2}; \biggl(\frac{1-r}{1+3r} \biggr)^{2} \biggr)-F \biggl(a,b;\frac {a+b-1}{2};1-r^{2} \biggr) \biggr] \\ &\quad=B(a,b)\lim_{r\rightarrow0^{+}} \biggl[ \biggl( \frac{\sqrt {8r(1+r)}}{1+3r} \biggr)^{1-a-b}F \biggl(\frac{b-a+1}{2}, \frac {a-b+1}{2};\frac{a+b+1}{2}; \biggl(\frac{1-r}{1+3r} \biggr)^{2} \biggr) \\ &\qquad{}-\frac{1}{2}r^{1-a-b}F \biggl( \frac{b-a+1}{2},\frac {a-b+1}{2};\frac{a+b+1}{2};1-r^{2} \biggr) \biggr] \\ &\quad=\frac{1}{2}B \biggl(\frac{a+b+1}{2},\frac{a+b-1}{2} \biggr)\lim_{r\rightarrow0^{+}} \biggl[2 \biggl(\frac{\sqrt{8r(1+r)}}{1+3r} \biggr)^{1-a-b}-r^{1-a-b} \biggr] \\ &\quad=-\infty. \end{aligned}$$

Let $x=x(r)=\sqrt{8r(1+r)}/(1+3r)$ and $x'=\sqrt{1-x^{2}}$. Then

3.12 $$ \frac{dx}{dr}=\frac{\sqrt{2}(1-r)}{\sqrt{r(1+r)}(1+3r)^{2}}=\frac {x'(1+3x')^{2}}{4x}. $$

Taking the derivative of $\phi(r)$ and using (3.12) leads to

3.13 $$\begin{aligned} \phi'(r)={}&-2\frac{\Gamma(\frac{a+b+1}{2})^{2}}{\Gamma(a+b)} \frac {1}{x^{a+b}x^{\prime a+b+1}F(a,b;\frac{a+b+1}{2};x^{2})^{2}}\cdot\frac {x'(1+3x')^{2}}{4x} \\ &{}+\frac{\Gamma(\frac{a+b+1}{2})^{2}}{\Gamma(a+b)}\frac {1}{r^{a+b}r^{\prime a+b+1}F(a,b;\frac{a+b+1}{2};r^{2})^{2}} \\ ={}&\frac{\Gamma(\frac{a+b+1}{2})^{2}}{\Gamma(a+b)}\frac {(1+3x')^{2}}{2(1+3r)x^{a+b+1}x^{\prime a+b}F(a,b;\frac{a+b+1}{2};x^{2})^{2}} \\ &{}\times \biggl[\frac{(xx')^{a+b-1}F(a,b;\frac {a+b+1}{2};x^{2})^{2}}{(rr')^{a+b-1}F(a,b;\frac {a+b+1}{2};r^{2})^{2}}-(1+3r) \biggr]. \end{aligned}$$

Therefore, the monotonicity of $\phi(r)$ follows immediately from (2.19) and (3.13). This, in conjunction with (3.11), gives rise to the desired result. □

## Results and discussion

In the article, we establish several quadratic transformation inequalities for Gaussian hypergeometric function $_{2}F_{1}(a,b;(a+b+1)/2;x)$ $(0< x<1)$. As applications, we provide the analogs of duplication inequalities for the generalized Grötzsch ring function

$$ \mu_{a,b}(r)=\frac{B(a,b)}{2}\frac{ F (a,b;(a+b+1)/2;1-r^{2} )}{F (a,b;(a+b+1)/2;r^{2} )} $$

introduced in [33].

## Conclusion

We find several quadratic transformation inequalities for the Gaussian hypergeometric function and Grötzsch ring function. Our approach may have further applications in the theory of special functions.
